# An Evaluation of the Content Quality, Readability, and Reliability of Publicly Available Web-Based Information on Pneumothorax Surgery in Ireland

**DOI:** 10.7759/cureus.63800

**Published:** 2024-07-04

**Authors:** Martin P Ho, Samin Abrar, Patrick P Higgins, Kishore K Doddakula

**Affiliations:** 1 Department of Cardiothoracic Surgery, Cork University Hospital, Cork, IRL

**Keywords:** web-based information, reliability, readability, pleurodesis, pleurectomy, pneumothorax

## Abstract

Introduction

The internet is increasingly the first port of call for patients introduced to new treatments. Unfortunately, many websites are of poor quality, thereby limiting patients’ ability to make informed health decisions. Within thoracic surgery, the treatment options for pneumothoraces may be less intuitive for patients to understand compared to procedures such as lobectomies and wedge resections. Therefore, patients must receive high-quality information to make informed treatment decisions. No study to date has evaluated online information regarding pneumothorax surgery. Knowledge regarding the same may allow physicians to recommend appropriate websites to patients and supplement remaining knowledge gaps.

Objective

This study aims to evaluate the content, readability, and reliability of online information regarding pneumothorax surgery.

Methods

A total of 11 search terms including "pneumothorax surgery," "pleurectomy," and "pleurodesis" were each entered into Google, Bing, and Yahoo. The top 20 websites found through each search were screened, yielding 660 websites.

Only free websites designed for patient consumption that provided information on pneumothorax surgery were included. This criterion excluded 581 websites, leaving 79 websites to be evaluated.

To evaluate website reliability, the Journal of American Medical Association (JAMA) and DISCERN benchmark criteria were applied. To evaluate the readability, 10 standardized tools were utilized including the Flesch-Kincaid Reading Ease Score. To evaluate website content, a novel, self-designed 10-part questionnaire was utilized to assess whether information deemed essential by the authors was included. It evaluated whether websites comprehensively described the surgery process for patients, including pre- and post-operative care. Website authorship and year of publication were also noted.

Results

The mean JAMA score was 1.69 ± 1.29 out of 4, with only nine websites achieving all four reliability criteria. The median readability score was 13.42 (IQR: 11.48-16.23), which corresponded to a 13th-14th school grade standard. Only four websites were written at a sixth-grade reading level. In the novel content questionnaire, 31.6% of websites (n = 25) did not mention any side effects of pneumothorax surgery. Similarly, 39.2% (n = 31) did not mention alternative treatment options. There was no correlation between the date of website update and JAMA (r = 0.158, p = 0.123), DISCERN (r = 0.098, p = 0.341), or readability (r = 0.053, p = 0.606) scores.

Conclusion

Most websites were written above the sixth-grade reading level, as recommended by the US Department of Health and Human Services. Furthermore, the exclusion of essential information regarding pneumothorax surgery from websites highlights the current gaps in online information. These findings emphasize the need to create and disseminate comprehensive, reliable websites on pneumothorax surgery that enable patients to make informed health decisions.

## Introduction

The internet is often the first port of call for patients exploring new diseases and treatment options [1,2]. However, the quality of web-based health information is often unregulated and at risk of lacking appropriate readability, reliability, and content.

Previous studies [3,4] have indicated that online health materials are written beyond the sixth-grade reading level recommended by the United States Department of Health and Human Services [5]. This may lead to poor health literacy among patients from less educated backgrounds. The readability of online health materials can be assessed using several validated readability tools [6].

Previous studies evaluating web-based information on stoma bags and pneumothoraces have indicated that the reliability of this information is poor [7-9], with the average website meeting only half of the Journal of the American Medical Association (JAMA) Benchmark reliability criteria [10]. Website reliability may also be assessed using other validated tools such as the DISCERN instrument [11].

To evaluate website content, several studies have designed novel content-scoring criteria for medical conditions including diabetic retinopathy and stoma bags [8,12]. However, these criteria are disease-specific and therefore cannot be extrapolated or compared with other studies. No broadly applicable, validated content criterion is available in the literature. Therefore, it is difficult to compare the content quality of online health literature.

This is a rare study that has attempted to evaluate web-based patient information on pneumothorax surgery. The objectives of this study are to quantitatively assess the readability, reliability, and content of the most encountered websites containing information on pneumothorax surgery.

This article was previously presented as a meeting abstract at the Sylvester O’ Halloran Perioperative Symposium on March 1, 2024. This article was previously presented as a poster at the 32nd Waterford Surgical October Meeting on October 14, 2023, the Irish Thoracic Society Scientific Meeting on November 10, 2023, the British Thoracic Society Winter Meeting on November 22, 2023, the Irish Surgical Training Group (ISTG) Aspiring Surgeons Research Symposium on January 13, 2024, the International Conference for Healthcare and Medical Students (ICHAMS) on February 16, 2024, and the Student Medical Summit on February 17, 2024.

## Materials and methods

Search strategy

A cross-sectional review of the most encountered websites concerning pneumothorax surgery was performed on June 10, 2023, at 10 am in Cork, Ireland, by two authors (M.H. and P.H). Both authors cleared their search history, cache, and cookies before the search.

The three most common search engines were used, namely Google, Bing, and Yahoo. These search engines encompassed over 95% of all internet searches in 2023 [13]. The first 20 websites encountered using each of 11 relevant search terms were identified: “Pneumothorax surgery,” “Pleurectomy,” “Pleurodesis,” “Collapsed lung surgery,” “VATS surgery,” “Chest drain surgery,” “Talc surgery,” “Tension pneumothorax surgery,” “Spontaneous pneumothorax surgery,” “Surgical emphysema surgery,” and “Air leak surgery.” This yielded 660 websites to be screened for inclusion.

Inclusion and exclusion criteria

Websites that were in English, free to access, contained information regarding pneumothorax surgery, and designed for public consumption (not for health professionals) were included.

Websites with paywalls, social media websites, paid adverts, video-only websites, and websites that did not discuss pneumothorax surgery were excluded.

Data collection

The following website characteristics were extracted from each website: the year in which the website was last updated and the authorship of the website (government-affiliated, academic, non-profit, media, or private). The entire website text was also extracted for readability, reliability, and content criterion analysis by two evaluators (M.H. and S.A.).

Readability

We utilized 10 online, freely available tools to assess website readability [6]: the Flesch-Kincaid Grade Level, the Flesch-Kincaid Reading Ease Score, the New Dale-Chall Score, the Spache Readability Score, the Simple Measure of Gobbledygook (SMOG) Index, the Coleman-Liau Index, the Gunning-Fog Score, the Automated Readability Index, the Linsear Write Score, and the Rix Score.

They analyzed elements of a text including word count and sentence length. They each yielded a numerical score that corresponded to a school grade reading level. Additionally, an overall median readability score for each website was included.

Reliability

We utilized two freely available, validated instruments (the JAMA Benchmark Criteria and the DISCERN instrument) to assess website reliability. Permission was not required to use these instruments.

The JAMA criteria (Table 1) evaluated the presence of four criteria to quantify website reliability. A score of +1 was given for each criterion fulfilled. This resulted in a score between 0 and 4 with a higher score corresponding to higher reliability.

**Table 1:** The Journal of the American Medical Association (JAMA) Benchmark Criteria questionnaire To evaluate website reliability, the author utilized the freely available Journal of the American Medical Association (JAMA) Benchmark Criteria [10]. Permission was not required to utilize this questionnaire. Please find the link to the original author’s website at https://jamanetwork.com/journals/jama/article-abstract/415407

Number	Does the website
1	Display the authorship including author affiliations and qualifications?
2	Display references, attributions, sources, and copyright information?
3	Display the date of the original posting and updates?
4	Disclose website ownership, sponsorship, advertising policy, and any potential conflicts of interest?

The DISCERN instrument (Table 2) consisted of 16 questions evaluating website reliability. Each question was scored on a scale from 1 to 5. A higher score of 5 indicated the quality criterion had been completely fulfilled, whereas a lower score of 1 indicated the criterion was not fulfilled at all. The maximum total score was 80.

**Table 2:** The DISCERN Instrument To evaluate website reliability, the author utilized the freely available DISCERN instrument [14]. Permission was not required to utilize this questionnaire. A link to the original author’s website is available at http://www.discern.org.uk/discern_instrument.php

Number	Questions	Score				
1	Are the aims clear?	1	2	3	4	5
2	Does it achieve its aims?	1	2	3	4	5
3	Is it relevant?	1	2	3	4	5
4	Is it clear what sources of information were used to compile the publication (other than the author or producer)?	1	2	3	4	5
5	Is it clear when the information used or reported in the publication was produced?	1	2	3	4	5
6	Is it balanced and unbiased?	1	2	3	4	5
7	Does it provide details of additional sources of support and information?	1	2	3	4	5
8	Does it refer to areas of uncertainty?	1	2	3	4	5
9	Does it describe how each treatment works?	1	2	3	4	5
10	Does it describe the benefits of each treatment?	1	2	3	4	5
11	Does it describe the risks of each treatment?	1	2	3	4	5
12	Does it describe what would happen if no treatment is used?	1	2	3	4	5
13	Does it describe how the treatment choices affect the overall quality of life?	1	2	3	4	5
14	Is it clear that there may be more than one possible treatment choice?	1	2	3	4	5
15	Does it provide support for shared decision-making?	1	2	3	4	5
16	Based on the answers to all of these questions, rate the overall quality of the publication as a source of information about treatment choices	1	2	3	4	5

Content

As there was no validated content criterion available in the literature, a novel, 10-part “Yes or No” questionnaire was designed by the authors (M.H. and P.H.) to evaluate website content (Table 3). It evaluated whether websites comprehensively described the surgery process for patients including pre- and post-operative care. The questionnaire can be modified to evaluate the content of any website examining surgical procedures.

**Table 3:** Self-designed 10-part content questionnaire

Number	Does the website
1	Provide background information on pneumothorax?
2	Describe what pneumothorax surgery entails?
3	Mention alternative treatment options (including no treatment at all)?
4	Mention the benefits/rationale of pneumothorax surgery?
5	Mention side effects/complications of pneumothorax surgery?
6	Provide pre-operative instructions?
7	Provide post-operative instructions?
8	Provide post-operative information (such as hospital stay length/recovery process)?
9	Instruct patients to ask their doctor/clinician if they have any questions?
10	Provide contact information for a patient to contact if they have any questions?

Statistical analysis

Website evaluation was performed by two authors (M.H. and S.A.). All statistical analysis was performed using Statistical Package for the Social Sciences (SPSS) version 28 (IBM Corp., Armonk, NY). Data was described using descriptive statistics (number, percentage, median, mean, and standard deviation). Pearson’s correlation coefficient was used to determine the association between questionnaire scores and the date of update of the website. A one-way ANOVA test was used to quantify the association between website authorship category and readability/reliability scores. Interobserver reproducibility was measured using the interclass correlation coefficient.

## Results

Authorship

**Figure 1:**
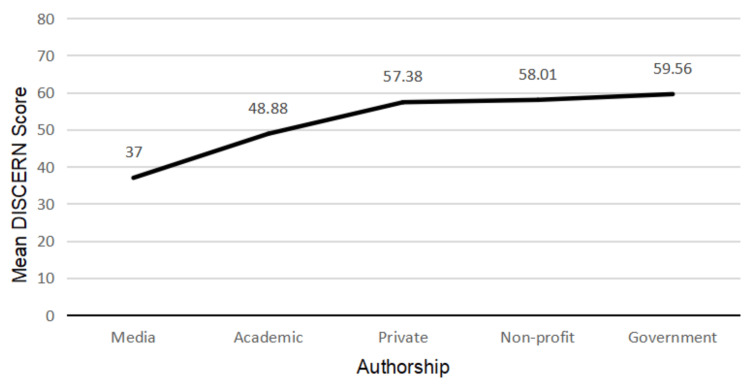
The mean DISCERN scores of websites from each authorship category

In total, 32 (40.5%) websites were private, 34 (43%) were non-profit, eight (10.1%) were government-affiliated, four (5.1%) were academic, and one (1.3%) was media. There was a statistically significant difference in the DISCERN scores between websites of different authorships (p = 0.007) (Figure 1). This was not seen in the JAMA (p = 0.197) or readability (p = 0.252) scores.

Readability analysis

The overall median readability score for all websites was 13.42 (IQR: 11.48-16.23). This corresponded to a 13-14th school grade or a first-year university student's reading level. Four (5.16%) websites were written at the sixth-grade reading level recommended by the United States Department of Health and Human Services. Of the 10 readability tools used, six of them rated the mean readability scores of websites as being at least “fairly difficult to read” (Table 4).

**Table 4:** Mean readability scores and comprehension level required

Readability tool	Mean (±SD)	Comprehension
Flesch-Kincaid grade level	10.07 (±2.41)	Fairly difficult to read
Flesch-Kincaid Reading Ease	52.52 (±13.69)	Fairly difficult to read
New Dale-Chall Score	14.02 (±2.46)	Very difficult to read
Spache Readability Score	4.59 (±0.67)	Scores above 3 are considered more difficult to read
Simple measure of Gobbledygook index	12.77 (±1.99)	Fairly easy to read
Coleman-Liau Index	11.38 (±2.11)	Fairly difficult to read
Gunning-Fog Score	13.71 (±2.77)	Difficult to read
Automated Readability Index	10.17 (±2.55)	Age 15-16
Linsear Write Score	13.15 (±8.08)	Suitable for someone with around 13 years of education
Rix	8.09 (±6.02)	Requires at least an eighth-grade education

Reliability analysis

We utilized the JAMA Benchmark Criteria and the DISCERN instrument to evaluate website reliability.

The mean JAMA reliability score was 1.69 (±1.29) out of 4. In total, 17 websites did not fulfill any of the JAMA criteria, while nine websites fulfilled all four criteria (Table 5). Notably, 50 (63.39%) websites did not display attributions/references. The interobserver reproducibility was r = 0.592 (p < 0.001).

**Table 5:** Number of JAMA criteria met by websites JAMA: Journal of the American Medical Association Benchmark Criteria.

JAMA Criteria met	n = (websites)	%
0/4	17	21.5
1/4	23	29.1
2/4	15	19
3/4	15	19
4/4	9	11.4

DISCERN

The mean DISCERN reliability score was 57.18 (±10.47) out of 80. The lowest-scoring website scored 28/80. The two highest-scoring websites were each 80/80. These were: http://thoracic.org/patients/patient-resources/resources/spontaneous-pneumothorax.pdf and http://europeanlung.org/en/information-hub/lung-conditions/primary-spontaneous-pneumothorax-psp/.

Notably, the weakest performing section within the questionnaire was, “Does it describe what would happen if no treatment is used?,” with a mean score of 2.48 (±1.55). The interobserver reproducibility was r = 0.387 (p = 0.017).

Content analysis

The mean number of content criteria met was 7 (±2.18) out of 10. Notably, 31.6% of websites did not mention side effects/complications of surgery, while 39.2% of websites did not mention alternative treatment options (Table 6). The interobserver reproducibility was r = 0.601 (p < 0.001).

**Table 6:** The percentage of yes/no responses to each content question

Number	Does the website	% Yes	% No
1	Provide background information on pneumothorax?	73.4	26.6
2	Describe what pneumothorax surgery entails?	89.2	10.8
3	Mention alternative treatment options (including no treatment at all)?	60.8	39.2
4	Mention the benefits/rationale of pneumothorax surgery?	89.2	10.8
5	Mention side effects/complications of pneumothorax surgery?	68.4	31.6
6	Provide pre-operative instructions?	51.3	48.7
7	Provide post-operative instructions?	63.9	36.1
8	Provide post-operative information (such as hospital stay length/recovery process)?	70.9	29.1
9	Instruct patients to ask their doctor/clinician if they have any questions?	74.7	25.3
10	Provide contact information for a patient to contact if they have any questions?	53.2	46.8

Website year of update

There was no correlation between the date of website update and JAMA (r = 0.158, p = 0.123), DISCERN (r = 0.098, p = 0.341), or readability (r = 0.053, p = 0.606) scores.

## Discussion

The internet has become an accessible tool for patients who wish to access information regarding diseases and treatment options. This study represents the first and most comprehensive evaluation of web-based information concerning pneumothorax surgery to date. A total of 660 websites were identified and 79 were evaluated.

The overall median readability score of our websites corresponded to a 13-14th school grade reading level, which is beyond the reading ability of the general population [15]. Only four websites were written at the sixth-grade reading level recommended by the United States Department of Health and Human Services [3,4]. Furthermore, six of the 10 individual readability tools used rated the mean readability scores of websites to be at least “fairly difficult to read”. Previous studies have shown websites concerning pancreatic cancer and diabetic retinopathy [12,16] to also be written above this recommended level. This indicates that patients do not understand a substantial portion of the information they are reading. This may reflect a wider trend of clinician authors not explaining concepts in a language familiar to patients. Websites should be written in a simple, understandable manner to not confuse patients further, thus facilitating informed decision-making.

Our reliability analysis showed that the mean JAMA score of our websites was 1.69 (±1.29) out of 4, with 63.3% of websites not displaying references. This concerning lack of website source transparency hinders the patients’ ability to make informed health decisions and potentially exposes the reader to misinformation. Additionally, our JAMA scores were lower than those from previous research evaluating web-based stoma information [8]. However, our mean DISCERN score for websites was 57.18 (±10.47) out of 80, which was slightly higher than the same study evaluating web-based stoma information [8]. These inconsistent results highlight the current challenges in accurately quantifying the reliability of web-based health information and reinforce the need for standardization of these types of questionnaires. To improve website reliability, search engines may recommend reliability checklists for websites. Completing these checklists could enhance the site's visibility.

The mean number of content criteria met was 7 (±2.18) out of 10 with many websites omitting important information regarding pneumothorax surgery. These included not mentioning the side effects of surgery (31.6% of websites) and not mentioning alternative treatment options (39.2% of websites). This may have occurred because website authors assumed that patients were already informed about these matters by their doctor. Nevertheless, these results are concerning, as an apparent lack of alternative treatment options may impact a patient’s ability to give informed consent. We designed this novel content criterion because we could not find an existing questionnaire that could successfully evaluate the content of websites concerning pneumothorax surgery. As a result, this questionnaire was not previously validated, and the results cannot be directly compared with previous studies that have created content questionnaires. However, it may be modified and contributed to a future validated content questionnaire that applies to all websites examining surgical procedures.

Limitations

Our study had several limitations. First, the study excluded video platforms such as YouTube and social media due to the complexity of analyzing several information modalities within a single study. These sites represent a significant source of patient health information [17]. Furthermore, this study only evaluated results from the top three search engines, thereby excluding all other search engines and a potential subset of patients who use these search engines. However, the search engines used make up 95% of all internet searches.

Additionally, despite our efforts to be comprehensive with our search strategy by including both esoteric and patient-friendly search terms, our search likely missed several websites. Furthermore, while search engines generally prioritize newer websites within the search engine algorithm, the authors did not exclude older websites with potentially outdated information.

Moreover, the DISCERN instrument was subjectively scored on a “one to five” scale, thus risking evaluator bias in the results. To mitigate this, two authors analyzed websites, and only the mean aggregate scores were included in the results. Similarly, two authors conducted the search strategy and applied the inclusion/exclusion criteria. However, due to the subjectivity of the DISCERN questionnaire, the interclass correlation coefficient was low (r = 0.387). This may limit the reliability of this section of the results. This was seen to a lesser extent with the JAMA (r = 0.592) and the content analysis (r = 0.601) as these were “Yes or No” questions. Refinement and standardization of the original DISCERN instrument may enhance the consistency of future studies’ results.

## Conclusions

This is one of the rare studies available in the literature to evaluate web-based pneumothorax surgery information. Most websites were written beyond the comprehension of the general population, hindering effective patient education and decision-making. Furthermore, many websites failed to disclose key information regarding pneumothorax surgery and failed to include sources for their information. These findings underscore the need for comprehensive, reliable websites on pneumothorax surgery that enable patients to make informed health decisions.
